# Integrating Spread Dynamics and Economics of Timber Production to Manage Chinese Tallow Invasions in Southern U.S. Forestlands

**DOI:** 10.1371/journal.pone.0033877

**Published:** 2012-03-19

**Authors:** Hsiao-Hsuan Wang, William E. Grant, Jianbang Gan, William E. Rogers, Todd M. Swannack, Tomasz E. Koralewski, James H. Miller, John W. Taylor

**Affiliations:** 1 Department of Wildlife and Fisheries Sciences, Texas A&M University, College Station, Texas, United States of America; 2 Department of Ecosystem Science and Management, Texas A&M University, College Station, Texas, United States of America; 3 Southern Research Station, U.S. Forest Service, Auburn, Alabama, United States of America; 4 Southern Region Forest Health Protection, U.S. Forest Service, Atlanta, Georgia, United States of America; Universita' del Piemonte Orientale, Italy

## Abstract

Economic costs associated with the invasion of nonnative species are of global concern. We estimated expected costs of Chinese tallow (*Triadica sebifera* (L.) Small) invasions related to timber production in southern U.S. forestlands under different management strategies. Expected costs were confined to the value of timber production losses plus costs for search and control. We simulated management strategies including (1) no control (NC), and control beginning as soon as the percentage of invaded forest land exceeded (2) 60 (Low Control), (3) 25 (Medium Control), or (4) 0 (High Control) using a spatially-explicit, stochastic, bioeconomic model. With NC, simulated invasions spread northward and westward into Arkansas and along the Gulf of Mexico to occupy ≈1.2 million hectares within 20 years, with associated expected total costs increasing exponentially to ≈$300 million. With LC, MC, and HC, invaded areas reached ≈275, 34, and 2 thousand hectares after 20 years, respectively, with associated expected costs reaching ≈$400, $230, and $200 million. Complete eradication would not be cost-effective; the minimum expected total cost was achieved when control began as soon as the percentage of invaded land exceeded 5%. These results suggest the importance of early detection and control of Chinese tallow, and emphasize the importance of integrating spread dynamics and economics to manage invasive species.

## Introduction

The ecological and economic costs of invasive species have been extensively discussed and the future impacts to global sustainability are a source of great concern [1], [2], [3], while few analyses have been directed toward cost efficiency for management of specific species [4]. Costs are predicted to continually rise at an alarming rate with increased trade and travel, as well as climate change, facilitating exotic introductions and invasions [5], [6], [7]. Ecologists, land managers, and even policy makers are increasingly recognizing the need to understand the causes and consequences of introduced species invasions, as well as assess the practicality of pursuing ecologically and economically effective management efforts [8], [9].

Among the most aggressive and costly invaders of southern U.S. forests is Chinese tallow tree (*Triadica sebifera* (L.) Small, Euphorbiaceae, synonyms include *Sapium sebiferum* (L.) Roxb and *Stillingia sebifera* Willd.). Chinese tallow was first introduced into the United States in the late 1700's and repeated introductions have occurred throughout the southeastern US until the mid-1900's [10]. Because of the large amount of vegetable tallow found in the seed, the Foreign Plant Introduction Division of the USDA promoted Chinese tallow planting in Gulf Coast states to establish a local soap and candle industry from 1920 to 1940, and closely monitored its management [11]. It was also commonly promoted as an ornamental tree by the horticultural trade throughout the mid-1990s [12]. Currently, Chinese tallow has escaped from cultivated locations and spread aggressively from the Gulf Coast of Texas to the Atlantic Coast of North Carolina [12], [13], [14], [15]. While the seeds provide a food resource for overwintering birds, this facilitates dispersal [16], and coupled with rapid growth [12], [17], a lack of herbivory or disease pressure [18], [19], [20], [21], tolerance to a broad range of environmental conditions [22], [23], and alterations to the microbial composition of the soil following invasion [24], result in the replacement of abandoned agricultural fields, native coastal tallgrass prairies, and southern forestlands by Chinese tallow woodland thickets [25]. It has become the dominant woody sapling species in east Texas and Louisiana forests [26], [27], and recent hurricane damage and feral hog activity in both Louisiana and Texas appears to be hastening the invasion of Chinese tallow throughout many southern ecosystems [28], [29], [30], [31], which could have profound long-term ecological and economic consequences. Recently, Wang et al. projected the spread of Chinese tallow northward and westward as much as 300 km from its present distribution along the Gulf Coast of Texas and Louisiana, reaching the Louisiana-Arkansas border by the year 2023, and covering 7.5 percent of forestlands in eastern Texas and Louisiana [32]. Moreover, projections by Pattison and Mack suggest that Chinese tallow is capable of expanding 500 km northward along the Atlantic Coast from its current distribution in the southeastern United States [14].

The severity of Chinese tallow invasions calls for mitigation. However, spatially-explicit bioeconomic frameworks for invasive species are lacking, so cost-effective control strategies have yet to be determined. Here we evaluate the economic costs (pertinent only to timber production) of Chinese tallow invasions on southern U.S. forestlands under different control strategies based on the percentage of land presently invaded by Chinese tallow tree. We consider the costs of timber loss as a result of Chinese tallow invasions and the costs associated with searching for and controlling Chinese tallow in invaded areas. This information on costs of different control efforts should help government agencies and private landowners decide whether and when to initiate control measures to maintain timber production.

## Methods

### Bioeconomic model

We modified the biological invasion model of Wang et al. [32] to include projections of the expected total costs associated with invasion of Chinese tallow into southern U. S. forestlands. The model of Wang et al. [32] represents range expansion by invasive species as a function of three distinct processes: arrival, establishment, and dispersal. It is a spatially-explicit, agent-based, stochastic, simulation model, programmed in VB.Net© (Microsoft, 2003), consisting of 13,820 geo-referenced cells (agents). Each cell represents a 2,428 ha (4,927 m×4,927 m; 6,000-acre) plot of forestland in the southern U.S. This cell size corresponds to the spatial sampling intensity employed by the U.S. Forest Service to compile their Forest Inventory and Analysis (FIA) dataset [33]. The FIA dataset contains data from a network of permanent ground plots that are part of a national array of sampling areas designed as the Federal base sample. The site and vegetation data gathered on each plot serve to support and quantify the information associated with each 2,428-ha sampling unit. Characteristics of each cell include landscape features, climatic conditions, and forest conditions that collectively represent the habitat quality of the cell for Chinese tallow, as well as the percentage of the area within the cell currently occupied by Chinese tallow. Annual changes in the percentage of land occupied by Chinese tallow result from growth within the cell plus invasion from other cells:
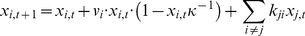
(1)where 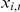 is the percentage of land occupied by Chinese tallow in cell 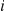 at time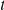; 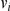 is the maximum spread rate within cell 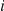; 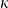 is the carrying capacity which we assume is 100% for all cells; and 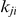 is a lognormal dispersal kernel which distributes the recruitment potential from cell 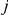 to cell 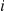 and varies with the invasion velocities. We assumed 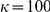 based on information in the nonnative invasive plant data set, which indicates that Chinese tallow already occupies over 95% of some FIA plots [34]. We also assumed 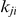 is a lognormal dispersal kernel because it has been used successfully to approximate the observed dispersal patterns for Chinese tallow [32] and a number of species with wind- and animal-dispersed seeds similar to Chinese tallow [35], [36], [37]. Simulation model dynamics were generated via iterative solutions of Eq. 1, with two additional rules: (1) cells within the dispersal kernel were invaded probabilistically, with the probability of invasion being equal to the volume within the two-dimensional normal distribution [38], [39], and (2) invasions could not originate from a cell until 3 years after its initial colonization (Chinese tallow do not produce seeds until age 3) [11]. See Wang et al. [32] for additional model details.

To include the economic impacts associated with invasion in the model, we represented expected total costs as a function of three components: damage costs (timber losses in $), searching costs, and control costs as:
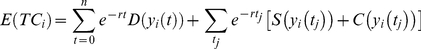
(2)where 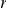 is the discount rate, 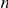 is the harvest cycle, 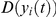 is the damage costs (timber losses in $) in cell 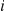 at time 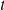, 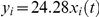, which is size (hectare) of the invaded area in cell 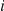 at time 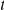, 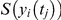 is the search costs in cell 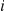 at time 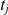, and 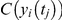 is the control costs in cell 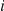 at time 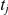. 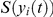 and 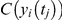 are greater than zero only when 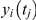 is above the control threshold at time 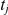 and 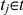. Choosing a discount rate generally has a large effect on the magnitude of the expected total cost [40]. Hence, we estimated costs using a range of discount rates (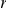) including 0.01, 0.03, 0.05, 0.07, and 0.09 encompassing those normally assumed and used in forestry-related benefit-cost analyses [41], [42], [43]. Because the timber industry usually harvests pine trees every 25 years and pulpwood trees every 15 years, with all other plant species also being removed at harvest via clear cutting [44], we assumed a harvest cycle (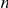) of 20 years on average.

We estimated damage costs as:
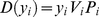
(3)where 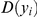 is the value of timber losses in cell 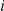, 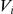 is the annual timber productivity without invasion (kg/ha) in cell 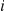, and 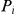 is the market price of timber ($/kg) in cell 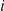. Timber losses are directly related to the land area occupied by the invasive species because the major impact of invasion is via forest stand replacement [25], [45], [46], [47], [48]. We estimated 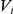 based on timber productivity data from USDA Forest Service data [33] (2008a, Fig. 1 and Table 1). The timber productivity 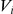 is a weighted average of the productivity of five major timber classes (pine sawtimber, pine chip-n-saw, pine pulpwood, mixed hardwood sawtimber, mixed hardwood pulpwood), and so is the stumpage price 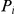, which was calculated using the data from TPO [49] and Timber Mart-South [44] (Table 2). The weights were based on the volume composition of the five major timber classes in the forest stand derived from the Forestry Inventory and Analysis database [33]. A cell can be re-invaded after being controlled.

**Figure 1:**
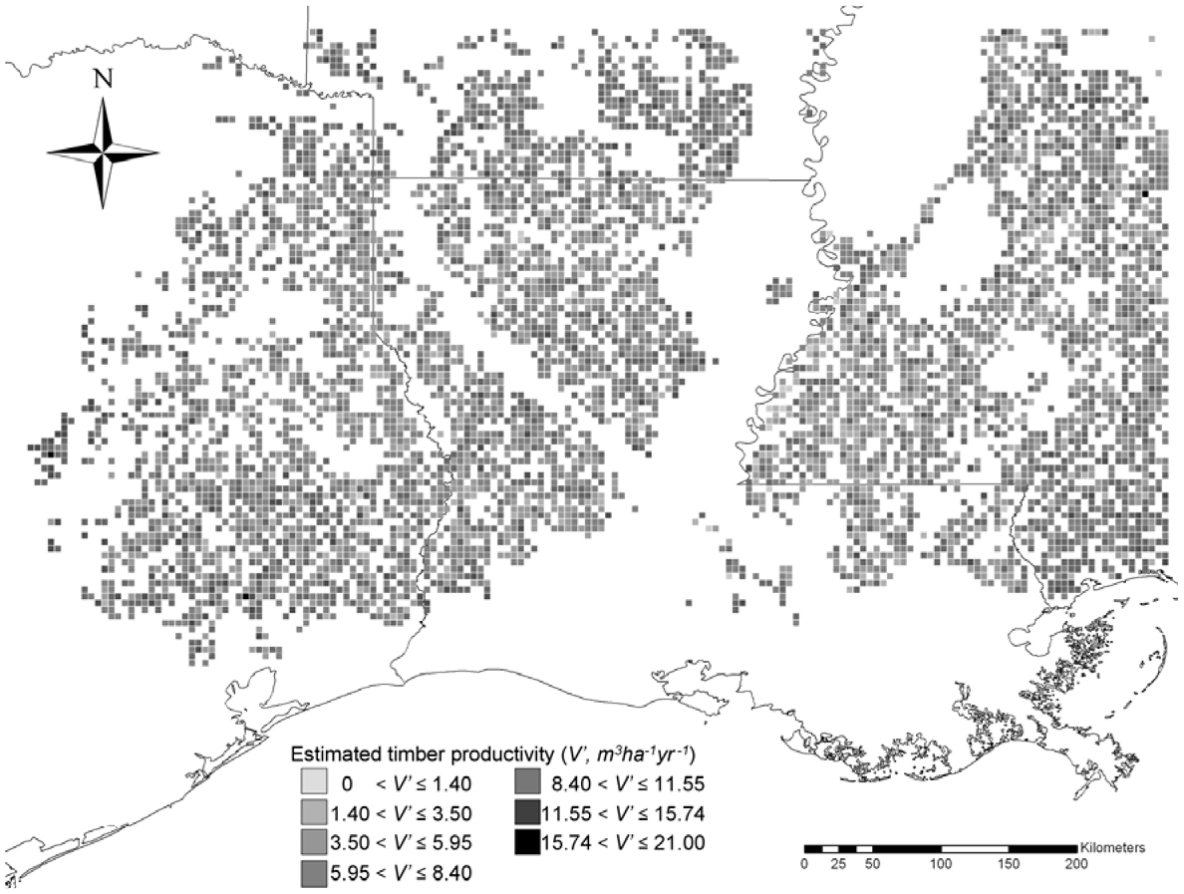
Estimated timber productivity (*V_i_*′, *cubic meter/hectare/year*) in southern U.S. forestlands [**33**].

**Table 1:** Estimated timber productivity (*V_i_*) in southern U.S. forestlands [33], [109].

Code	Estimated timber productivity (*V_i_*′; *cubic meter/hectare/year*)	Estimated timber productivity (*V_i_*; *kg/hectare/year*)^1^
1	15.74≤*V_i_*′<21.00	5536.98
2	11.55≤*V_i_*′<15.74	4102.30
3	8.40≤*V_i_*′<11.55	2981.45
4	5.95≤*V_i_*′<8.40	2152.03
5	3.50≤*V_i_*′<5.95	1412.27
6	1.40≤*V_i_*′<3.50	739.76
7	0≤*V_i_*′<1.40	201.75

1 We assumed that 907.18 kg (1 short ton) of wood has 3.02 cubic meters of solid wood [109].

**Table 2:** Summary of timber productivity in southern U.S. forestlands by timber class, indicating percentage of harvest [49], stumpage prices (averages from 2000 to 2008) [44], and market price, calculated as the weighted average.

Timber class	Percentage	Stumpage price (*dollars/kg*)	Market price (*P_i_*; *dollars/kg*)
Pine sawtimber	34.63	0.0405	
Pine chip-n-saw	18.02	0.0245	
Pine pulpwood	25.51	0.0077	0.0234
Mixed hardwood sawtimber	9.58	0.0223	
Mixed hardwood pulpwood	12.26	0.0065	

We estimated control and search costs based on phone interviews conducted from July 15 to 21, 2009 with personnel from major invasive plant control companies in the region [50]. Because invasion control services usually are requested after an invasion has been discovered, search costs seldom are estimated separately. However, we did obtain a search cost estimate of 19.77 $/ha (8 $/acre) from one company. While we obtained searching costs from only one company, we assumed searching costs were governed by the population size which determined whether it was easy or difficult to detect a current invasion. We assumed a cost of $48,000 ($19.77×2,428 ha) for searching 100% of a forest plot to detect an invasion of ≤1.00% of the area, and we decreased searching costs as the invaded area increased following Mehta et al. (2007) and Carrasco et al. (2010) [51], [52]:
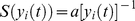
(4)thus, 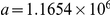; for 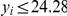 (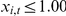) we assume 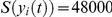.

We estimated control costs based on the information from Superior Forestry Service, Inc. (Tilly, AR), which provided the most detailed information and was licensed to conduct invasion control services in several states (Table 3). We assumed control costs increase exponentially as the percentage of invaded area increases:
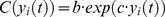
(5)and estimated 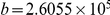 and 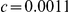 (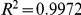) based on the information from Superior Forestry Service, Inc. in Table 3.

**Table 3:** Estimates of control costs (*dollars/hectare*) based on phone interviews with invasive plant control companies during the period from July 15 to 21, 2009.

Company			Control prices			
Marshfield Forest Service, Inc.^1^			$25–$988			
ChemPro Services, Inc.^1^			$371–$1235			
BASF-The Chemical Company^1^			$124–$988			
Progressive Solutions^2^	$25–$62 (1%–5%)	$62–$185 (5%–20%)	$185–$321 (20%–40%)	$321–$494 (40%–60%)	$494–$741 (60%–80%)	$741–$1112 (80%–100%)
Superior Forestry Service, Inc.^3^	$111–$210 (1%–25%)		$210–$457 (25%–60%)		$457–$1446 (60%–100%)	

1 General price range for controlling all invasive plant species.

2 Price ranges for controlling all invasive plant species depending on percentage of land invaded; an estimated searching cost of 19.77 *dollars/ha*.

3 Price ranges for controlling Chinese tallow depending on percentage of land invaded.

### Projection of costs

**Figure 2:**
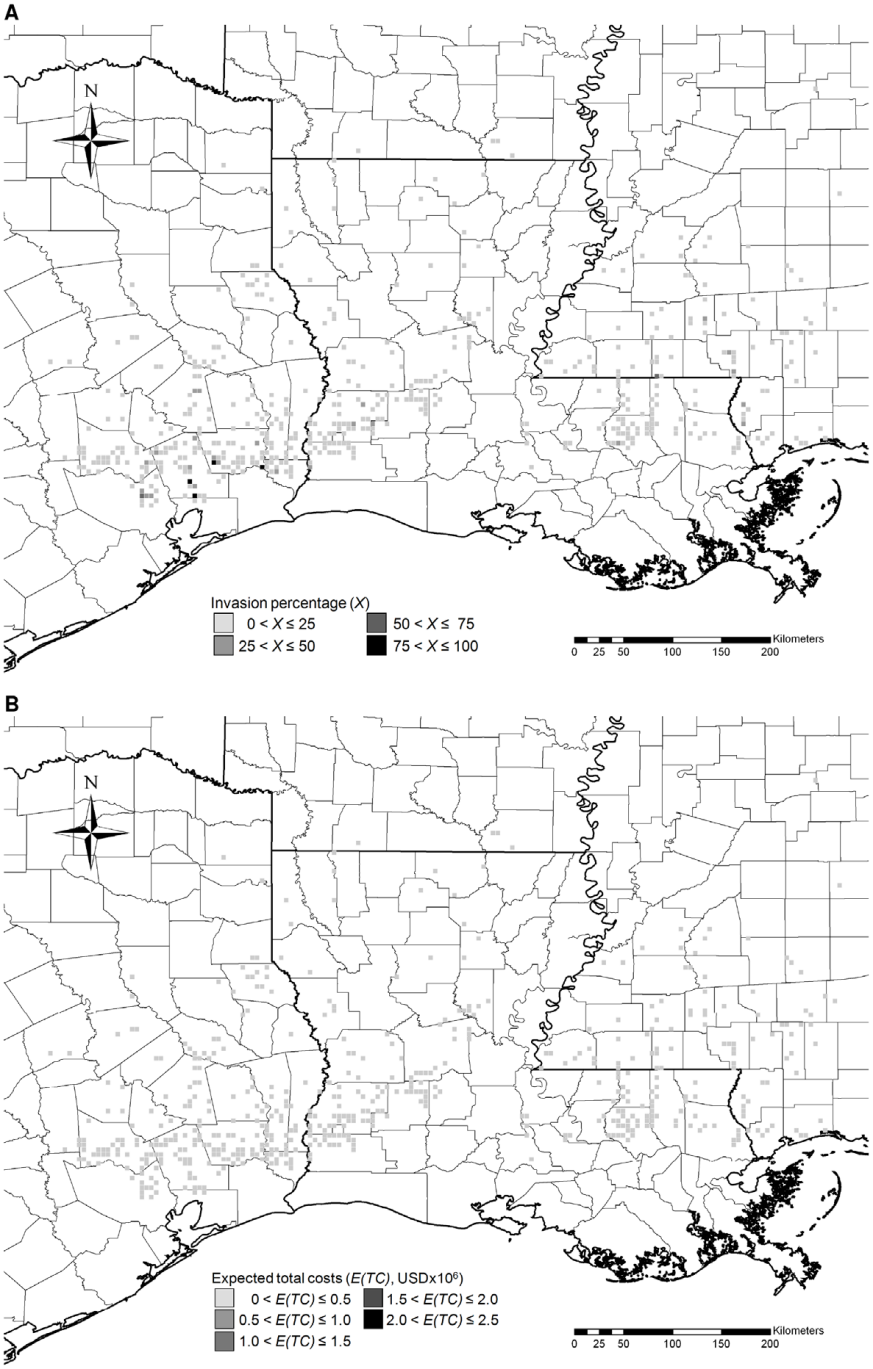
Observed pattern of Chinese tallow invasion (A) and associated expected total costs (B) in the year 2003 based on the nonnative invasive plant dataset [**53**].

To evaluate the economic costs of controlling Chinese tallow, we ran 240, 20-year, Monte Carlo simulations from observed pattern of Chinese tallow invasion in the year 2003 (Fig. 2) under each of four management scenarios: (1) no control (NC), and control beginning as soon as the percentage of land invaded exceeded (2) 60 (low control intensity, LC), (3) 25 (medium control intensity, MC), and (4) 0 (high control intensity or immediate control without delay, HC). These strategies were chosen to be generally representative of the broad range of potential management scenarios (indifferent, reactive, active, and proactive, respectively) available to agency personnel and private landowners. We defined the thresholds for low, medium, and high control intensities, and assigned the corresponding search and control costs based on the information from Superior Forestry Service, Inc., as represented in equations 4 and 5. We also ran simulations for different management scenarios besides LC, MC and HC to identify the control threshold that would minimize the expected total costs. We initialized each simulation with the percentage land cover of Chinese tallow reported in the latest Forest Service field sampling cycle [53]. Control decisions during simulations were made each year on a cell-by-cell basis. The percentage land cover of Chinese tallow in each cell meeting the control criterion was reduced to zero, and the discounted present values of damage, and search and control costs were recorded (see Eq. 2). We exported geo-referenced simulated data on land cover of Chinese tallow from VB.NET^©^ to Excel files and subsequently imported the Excel files into ArcView® to analyze spatial-temporal patterns of invasion.

### Data sources

To parameterize the Chinese tallow invasion model for eastern Texas, Louisiana, western Mississippi, and southern Arkansas, we obtained data on environmental and ecological characteristics of geo-referenced USDA Forest Service sample plots from the Forest Inventory and Analysis (FIA) dataset [33], and data on percentage land cover of Chinese tallow from the Non-native Invasive Plants dataset [53]. We then followed the procedure described in Wang et al. [32] to estimate Chinese tallow growth rates, dispersal velocities, and the resulting annual changes in percentage of land cover occupied by Chinese tallow

For cost estimation, we obtained data on timber productivity from the Forest Service Timber Product Output dataset [49], the Timber Mart-South dataset [44], and other USDA sources [33], [54]. Data on costs of controlling invasive species were derived from interviews with personnel from major invasive plant control companies in the region [50]. Based on these data, we estimated expected total costs as the sum of damage costs (loss of timber productivity), control costs, and search costs [51], [55], as described above in estimation of expected total costs.

## Results

**Figure 3:**
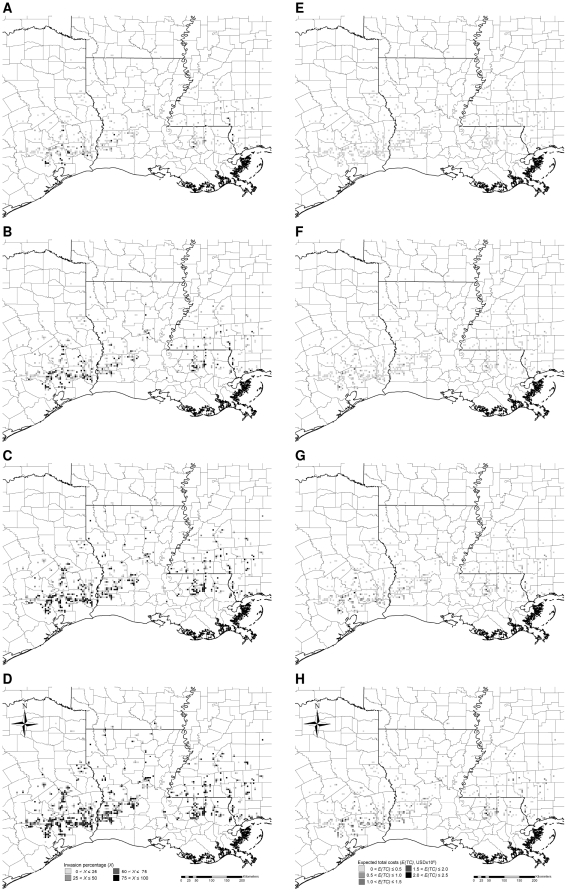
Typical simulated patterns of Chinese tallow invasion without control (A–D) and associated expected total costs (E–H) accumulated to the 5^th^, 10^th^, 15^th^, and 20^th^ year, respectively. Simulations were initialized with the observed pattern of Chinese tallow invasion in the year 2003 based on the nonnative invasive plant dataset [53] (see Fig. 2A). Each time series of patterns is based on one randomly-chosen stochastic simulation.

**Figure 4:**
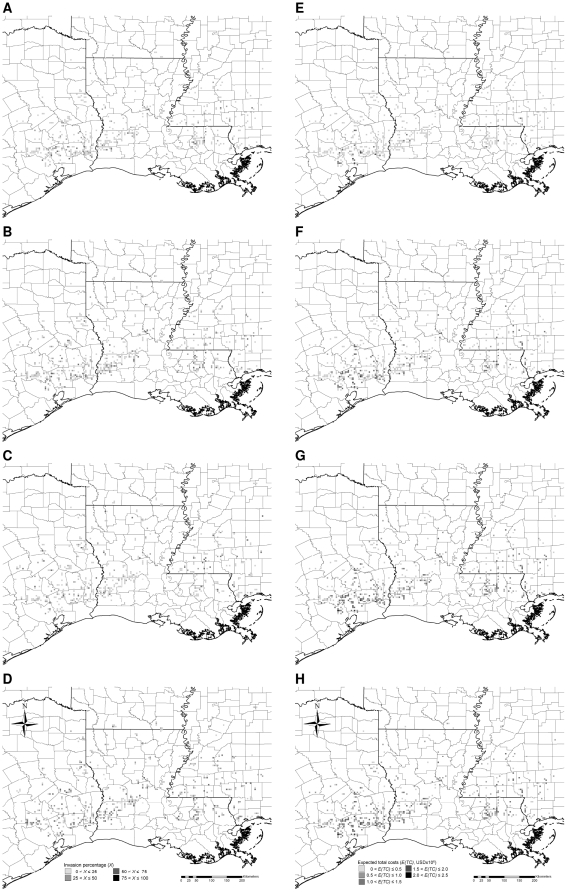
Typical simulated patterns of Chinese tallow invasion with low intensity control (A–D) and associated expected total costs (E–H) accumulated to the 5^th^, 10^th^, 15^th^, and 20^th^ year, respectively. Simulations were initialized with the observed pattern of Chinese tallow invasion in the year 2003 based on the nonnative invasive plant dataset [53] (see Fig. 2A). Each time series of patterns is based on one randomly-chosen stochastic simulation.

**Figure 5:**
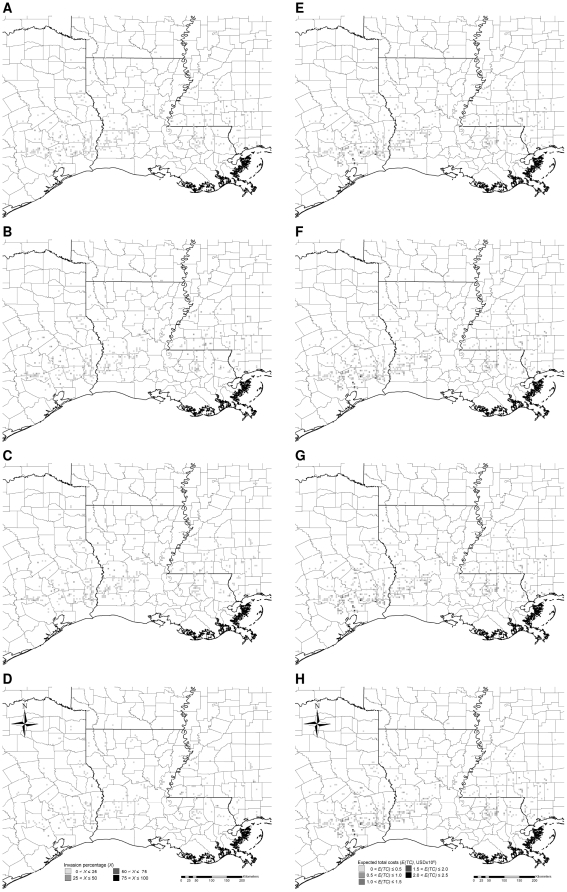
Typical simulated patterns of Chinese tallow invasion with medium intensity control (A–D) and associated expected total costs (E–H) accumulated to the 5^th^, 10^th^, 15^th^, and 20^th^ year, respectively. Simulations were initialized with the observed pattern of Chinese tallow invasion in the year 2003 based on the nonnative invasive plant dataset [53] (see Fig. 2A). Each time series of patterns is based on one randomly-chosen stochastic simulation.

**Figure 6:**
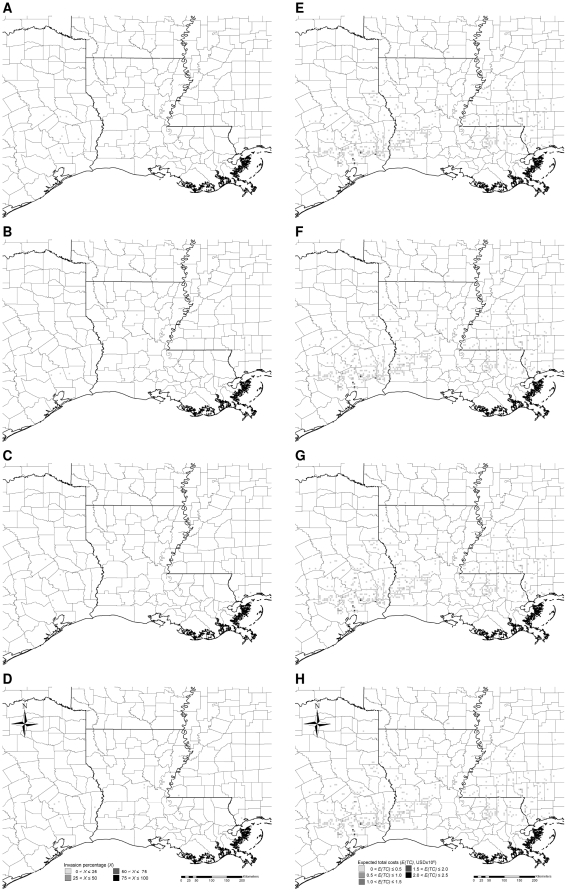
Typical simulated patterns of Chinese tallow invasion with high intensity control (A–D) and associated expected total costs (E–H) accumulated to the 5^th^, 10^th^, 15^th^, and 20^th^ year, respectively. Simulations were initialized with the observed pattern of Chinese tallow invasion in the year 2003 based on the nonnative invasive plant dataset [53] (see Fig. 2A). Each time series of patterns is based on one randomly-chosen stochastic simulation.

Simulated Chinese tallow invasions, if not controlled, spread northward and westward into the forests of Arkansas and colonized lands along the Gulf of Mexico within 20 years, with about 14% of all forest cells invaded to some extent, and >62% of the invaded cells exhibiting >50% of the land occupied by Chinese tallow (Figs. 3A–3D). Assuming a 5% discount rate, about 6% of the cells, most of them along the Gulf of Mexico, accumulated expected total costs >0.5 million USD over the 20-year period (Figs. 3E–3H). Under the low intensity control, invasions advanced more slowly, but still spread northward and westward into the forests of Arkansas, with approximately 7% of the cells invaded, and roughly 21% of the invaded cells exhibiting >50% occupancy by Chinese tallow (Figs. 4A–4D). About 10% of the cells, primarily in the southern half of Texas, Louisiana, and Mississippi, accumulated expected total costs >0.5 million USD (Figs. 4E–4H). Under the medium intensity control, invasions advanced more slowly, there were no severely-invaded areas (maximum invasion intensity <30%), with about 3% of the total forest area invaded, and roughly 12% of the invaded areas exhibiting >20% occupancy (Figs. 5A–5D). Only about 4% of the cells, primarily in southeastern Texas, accumulated expected total costs >0.5 million USD (Figs. 5E–5H). Under the high intensity control, invasions were limited to the southern most part of the study area, with <0.5% of the cells invaded, and with most invaded cells exhibiting <5% occupancy (Figs. 6A–6D). Barely 1% of the cells, primarily in southeastern Texas, accumulated expected total costs >0.5 million USD (Figs. 6E–6H). Ranking of the control strategies with regard to expected total costs was the same for discount rates ranging from 1 to 9%, although, of course, absolute dollar values increased with lower discount rates (Table 4). Hereafter we present all estimated costs based on a 5% discount rate.

**Table 4:** Expected total costs assuming discount rates (*r*) of 1%, 3%, 5%, 7%, and 9% accumulated over a 20-year period without control (NC), and with low (LC), medium (MC), and high (HC) intensity and expected total costs for the 5% discount rate also are divided into damage, searching, and control costs.

Accumulated costs (*million dollars*)	NC	LC	MC	HC
Expected total costs, *r* = 0.01	503.07	612.33	337.36	214.32
Expected total costs, *r* = 0.03	384.15	480.18	276.02	207.50
Expected total costs, *r* = 0.05	296.43	380.37	229.21	201.57
Expected total costs, *r* = 0.07	231.30	304.43	223.12	196.30
Expected total costs, *r* = 0.09	182.59	246.22	179.98	171.53
Damage costs	296.43	114.76	46.73	3.66
Searching costs	0.00	0.16	0.54	18.04
Control costs	0.00	265.45	181.94	179.87

**Figure 7:**
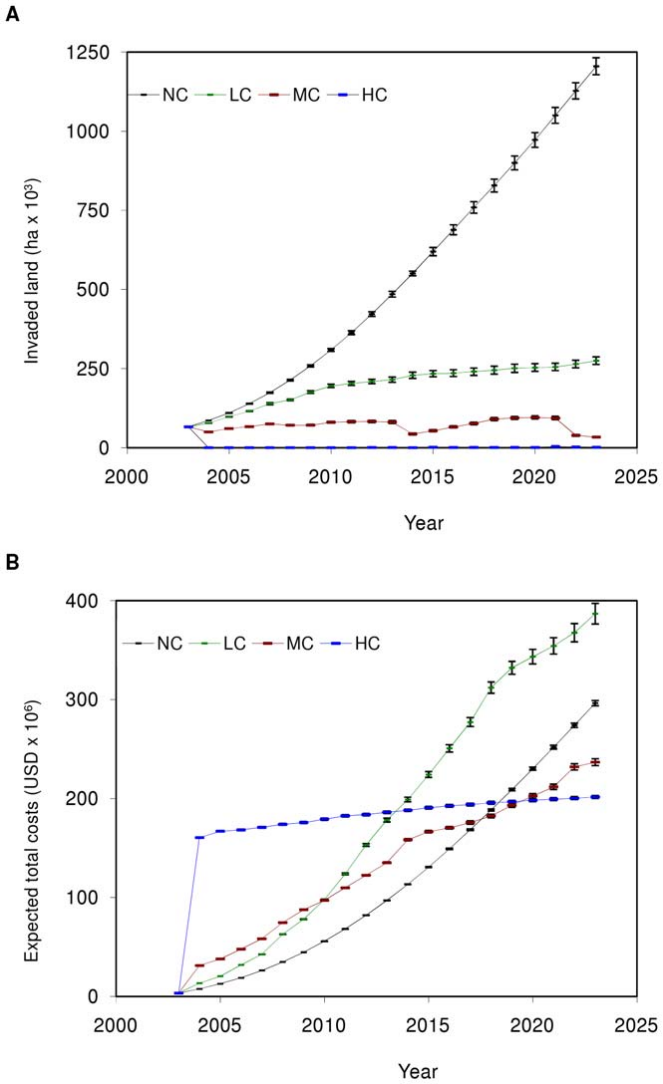
Mean area (± SE) invaded by Chinese tallow (A) and associated mean expected total costs (± SE) (B) without control (NC), and with low (LC), medium (MC), and high (HC) intensity control accumulated over a 20-year period.

Without control, both the total area invaded by Chinese tallow and the expected total costs increased exponentially, reaching ≈1.2 million hectares (Fig. 7A) and ≈$300 million (Fig. 7B, Table 4), respectively, within 20 years. Under the low intensity control, the invaded area increased in a roughly linear manner at a much slower rate, reaching almost 300 thousand hectares (Fig. 7A), however, the expected total costs were higher than those with no control, reaching almost $400 million (Fig. 7B, Table 4), with control and damage costs accounting for roughly 2/3 and 1/3 (Table 4), respectively, of total costs, and with negligible search costs. Under the medium intensity control, the invaded area was maintained relatively close to initial conditions (≈66 thousand hectare in the year 2003), with noticeable decreases in 2014 and 2022 (Fig. 7A). These decreases were due to increased control efforts necessitated by the control-induced synchronization of Chinese tallow re-invasion. The expected total costs increased in a roughly linear manner to ≈$230 million (Fig. 7B, Table 4), with slight decreases in the rate of increase in the years after the higher control efforts, due to the subsequent decrease in the need to control. Control and damage costs accounted for about 80% and 20% (Table 4), respectively, of total costs, again with negligible search costs. Under the high control intensity, Chinese tallow invasions were maintained below 4,100 hectares (Fig. 7A) with expected total costs increasing in a roughly linear manner to ≈$200 million (Fig. 7B, Table 4). Control, damage, and search costs accounted for ≈90%, 8%, and 2% (Table 4), respectively, of total costs.

**Figure 8:**
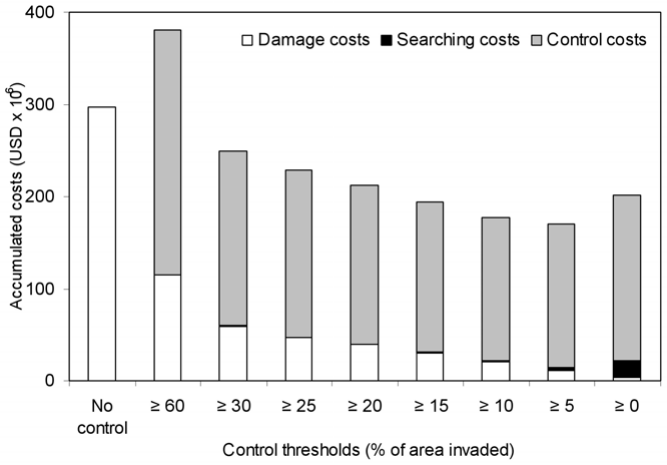
Mean expected total costs including damage, searching, and control costs, accumulated over a 20-year period with different control thresholds.

Results of the additional simulations suggested that a control threshold of 5% would minimize the expected total costs (Fig. 8). Decreases in damage costs were proportionally greater than decreases in control costs as the control threshold was decreased from 60% to 5%, and also were greater in absolute terms as the control threshold was decreased from 30% to 5%. Search costs increased roughly exponentially, but still were negligible under the 5% control threshold strategy. As the control threshold was decreased from 5% to 0 (immediate control upon encountering Chinese tallow), expected total costs increased, with search costs more than quadrupling, and being roughly equal, in absolute terms, to the increase in control costs.

## Discussion

We have presented a dynamic bioeconomic approach for managing the impact of invasive species range expansion which combines predictions of the spatial-temporal advance of a biological invasion with estimations of the concomitant timber losses and expected total economic costs. Our approach integrates invasion ecology and natural resource economics within a spatially explicit, agent-based, simulation framework to compare the efficacy of alternative invasion control scenarios. In the following sections, we consider the theoretical and empirical basis for our approach, interpret our findings in terms of the bioeconomic implications for Chinese tallow management, and provide some suggestions for future study.

### Theoretical and empirical basis for our approach

Invasive species have had enormous negative environmental and economic impacts worldwide [56], whether measured in terms of direct ecological impacts, loss of ecological services, economic damages, or costs of control [57]. Nonetheless, quantitative frameworks explicitly representing both dispersal capabilities and population growth of the invading species, as well as costs of both control efforts and economic losses due to damage to the resource are rare [58], [59]. Hence, we designed a model that represents both dispersal rate over the landscape and local rates of population growth, as well as current and accumulated economic damage and costs of control, including searching and control costs, at both local and landscape scales.

The dispersal of invasive species has been a subject of both theoretical and empirical study for decades [60], [61], [62], with academic roots extending back to the linear diffusion models of Fisher and Skellam [38], [63]. Subsequently, dispersal distances have been estimated using a variety of probability distributions [32], [64], [65], [66], [67], [68], [69]. Dispersal models have been both spatially-implicit and spatially-explicit depending on purpose [70]. Spatially-implicit models have been useful for species that reach high densities in part of their range and are essentially absent elsewhere [59], [71]. Spatially-explicit models have been useful for species that spread continuously into adjacent habitats or exhibit long-distance dispersal [72], or whose dispersal patterns are influenced significantly by landscape structure at the spatial scale of interest [73], [74]. Obviously, choosing the appropriate approach depends on the biological complexity of the predominant dispersal mechanisms involved, as well as the availability of data [75]. We represented dispersal using the lognormal probability distribution [32], implicitly denoting seed dispersal by both wind and animals via a spatially-explicit framework [16], [36], [37]. Lognormal probability distributions with source effects (peaked form) and path effects (fat-tailed form) have been used successfully to approximate observed dispersal patterns of a number of species with wind- (source effects) and animal- (path effects) dispersed seeds similar to Chinese tallow [35], [36], [37], [76], [77]. Our model projected the spread of Chinese tallow from the Gulf of Mexico northward and westward into the forests of Arkansas at a velocity of approximately 1200 m per year (Fig. 3A–3D), which is similar to the empirically-estimated dispersal velocity of approximately 1000 m per year reported by Renne et al. [16] based on experiments involving Chinese tallow seed dispersal by birds in coastal South Carolina. To date, we have developed similar models for Chinese privet (*Ligustrum sinense* Lour.), European privet (*Ligustrum vulgare* L.) and Japanese honeysuckle (*Lonicera japonica* Thunb) [50], and a similar approach also has applied successfully to ragweed (*Ambrosia artemisiifolia* L.) in Austria [78]. Nonetheless, exploration of different dispersal mechanisms using maximum likelihood methods still remains a fruitful area of investigation for Chinese tallow.

It should be noted that had we assumed a different probability distribution to estimate dispersal distances, our projected dispersal velocities and the resulting economic costs would have been different. Had we chosen a fat-tailed distribution other than the lognormal (e.g. geometric, half-Cauchy, or 2Dt), projected velocities and costs would have been similar since fat-tail distributions generate long dispersal distances. Had we chosen a thin-tailed distribution (e.g. exponential power, Weibull, or Laplace), projected velocities would have been slower and projected costs would have been smaller since thin-tail distributions generate short dispersal distances.

The post-invasion growth of invasive species also has been a subject of much study, and population growth models have been firmly rooted in population dynamics theory (Verhulst 1838, Lotka 1925, Volterra 1926), using both exponential and density-dependent growth models depending on purpose [51], [56]. Such models often are spatially-implicit and have been most useful when local impacts of invasion are of primary interest, or in situations in which spatial relationships are otherwise considered unimportant, such as fish invasions [79]. Certainly the population perspective will remain a cornerstone for the evaluation of control strategies for isolated invasions [59]. We designed local population growth using a density-dependent model with growth rate also a function of local habitat quality, reflecting habitat heterogeneity at the landscape level [32]. The resulting projections of post-invasion growth indicated that in favorable habitat 99% local (within cell) occupancy would be reached in 18 years, and complete occupancy in 29 years, after initial colonization [32], which is similar to the local spread rate reported by Bruce et al. for favorable habitats (20–30 years) [25].

Identification of cost-efficient control strategies for invasive species ideally would be based on estimates of both the costs of control efforts and the economic losses due to damage to the resource [59], [80]. Control costs often can be collected relatively easily from markets [81], although the effectiveness of control may be uncertain and may vary depending on severity of invasion [80], [82], and there may be non-target effects [83]. We calculated local control costs based on a range of estimates of local invasion severities provided by several invasion control companies [50]. Unfortunately, since land-owners commonly hire invasion control companies to execute control rather than search for signs of invasion, we could only obtain searching costs from one company. Based on this information, and the logic that it is relatively easier to detect invasion when the local level of invasion is large, we represented local searching costs as a decreasing function of the percentage of the local area invaded [51], [52]. While previous studies have used a variety of approaches to estimate the overall magnitude of potential costs [1], [84], [85], few, if any, have estimated control and searching costs separately [57], [86].

Of course, had we assumed a different functional form of the relationship between area invaded and searching costs, we would have obtained different estimates of expected total costs. Had we assumed searching costs increase exponentially with decreasing invaded area [87], the relative differences in total costs among treatments would not have changed but the absolute total costs of each treatment would have been greater. Had we assumed searching costs decrease linearly with decreasing invaded area [88], total costs under the high control intensity treatment, which is initiated when >0 percent of the area is invaded, would have decreased and total costs under the low control intensity treatment, which is initiated when >60 percent of the area is invaded, would have increased. If the searching cost per unit area were large enough this could have made the low control intensity treatment the most costly. Obviously, more work is needed to relate searching costs to area invaded.

Economic losses due to resource damage also are problematic to estimate because they include the impact of invasion on nonmarket as well as market values. A straightforward monetary measure of the impact of invasion on market values of many agricultural products is the average value of the product lost due to invasion damage [57]. There is no simple measure of the impact of invasion on nonmarket values because this may include several different kinds of ecosystem services, such as landscape aesthetics and altered fire regimes, among others [84], [86]. Hence, we calculated local economic damage to the resource using current monetary values from the resource (timber) market [44], as well as local resource (timber) productivity [49].

Our aim in the present study is to provide spatially-explicit, temporally-dynamic, representations of the economic aspects of biological invasions. Most invasive plant management, including that of Chinese tallow, has emphasized controlling either highly-infested areas or areas in the early stages of recruitment and establishment [89], which, from an economic perspective, is not necessarily ideal due to the trade-off between the costs of control efforts and the economic losses from resource damage. This emphasizes the need for spatially-explicit, temporally-dynamic models to suggest where and when (1) effective monitoring and/or control plots might be placed, (2) initial invasions might be expected, (3) invasions might affect highly-productive areas, and (4) estimated total costs of control might exceed the avoided loss of timber production. While previous models have projected spatial patterns of the biological aspects of invasion over time under different control strategies [72], [74], to the best of our knowledge, our model is the first to project spatial patterns over time of the economic aspects of invasion under different control strategies.

### Bioeconomic implications for Chinese tallow management

Our simulation results re-emphasized the importance of early detection and proactive control of Chinese tallow invasions in southern U.S. forestlands, a finding that echoes the almost universal conclusion of invasion studies focused not only on woody plants (e.g., Cacho et al. [80]) but also on herbaceous plants (e.g., Regan et al. [90]) and insects (e.g., Liebhold and Bascompte, El-Sayed et al. [91], [92]). Early control from a biological perspective is, of course, ideal. From an economic perspective, particularly for woody invaders, per-unit control costs typically increase markedly with invaded area past the threshold at which it is necessary to use mechanical methods [50], [51], [52]. This appears to be the case for Chinese tallow (Table 3). Our simulations indicated the minimum expected total cost occurred at a 5% invasion control threshold (Fig. 5). If control was initiated when <5% of the area was invaded, the reduced damage costs were not enough to offset the increased search costs. Thus eradication, which often is the presumed goal of invasion control [74] since it avoids long-term control costs [55], was not the most cost-efficient control strategy for our simulated Chinese tallow invasions. Burnett et al. [93] also recommended postponing control efforts for the velvet tree (*Miconia calvescens*) on the islands of Oahu and Molokai in Hawaii until 1400 and 2300 trees were found, respectively. If control was initiated when >5% of the area was invaded, overall total costs were markedly higher due to additional control costs and greater damage costs. Sharov and Liebhold [71] found that overall total costs for controlling gypsy moths (*Lymantria dispar*) in the southern U.S. increased when the distance from the invasion front to the end of the uninfested area becomes <200 km.

Currently, Chinese tallow occupies >5% of the area in ≈26% of the infested forestlands in our study area that it has invaded [53], and few forestland owners in areas susceptible to Chinese tallow invasions have initiated aggressive control measures. The limited efforts being pursued are akin to the low intensity control scenario simulated in this study (control initiated when 60% of the invaded area is occupied by Chinese tallow). Although from a biological perspective simulated low intensity control decreased the total extent of the invasion by >77% (Fig. 4A–4D), from the economic perspective of expected total costs it was the worst management scenario (Fig. 4E–4H). Our simulations suggested that at least medium intensity control (control initiated when 25% of the invaded area is occupied by Chinese tallow) would be needed to decrease the annual expected total costs associated with persistent propagule pressure and continual establishment of invasive seedlings. Such intensified control efforts would require coordination at the regional level.

### Suggestions for future study

Providing useful predictions of the rate of spread of biological invasions and their associated economic costs remains a challenge which is of global concern [1], [2], [3]. The challenge arises in large part because bioeconomic factors affecting the cost-efficiency of invasion control operate at different spatial and temporal scales. Ecologically, habitat quality affects shorter-term population growth and local spread of invasive species, while landscape characteristics interact with innate dispersal abilities of invaders to affect longer-term regional spread [94], [95], [96]. Economically, expense of current methods affect shorter-term cost-effectiveness of control [74], [90], while general economic trends affect longer-term total costs imposed by invasion [40], [55]. Wang et al. [32] recently discussed the ecological basis for our approach for predicting the rate of spread of biological invasions by terrestrial plants. Below we discuss some bioeconomic considerations regarding our representations of damage, search, and control costs, and provide suggestions for future improvements.

We estimated damage costs based on data on timber productivity [33], [49] and stumpage prices [44] and incorporated this relationship (Eq. 3) into the biological invasion model of Wang et al. [32]. Such an approach obviously underestimates damage costs because invasive species not only decrease forest productivity [48], [97], but also degrade diversity and wildlife habitat [98], [99], alter ecosystem structure [100], [101], function [102], [103], and disturbance regimes [104], as well as hinder forest use and management [105]. In addition to the difficulties associated with representation of these ecological and social damage costs, the interaction of stochastic fluctuations in environmental and economic conditions produce variability in economic damage costs across time [59]. Given appropriate variability estimates, we easily could incorporate a stochastic representation of economic damage costs into our model, however, the development of appropriate methods (both market and non-market techniques) to estimate the uncertainty associated with these damages remains an area of active research [57], [81].

We estimated search and control costs based on recent information from invasion control companies [50] and incorporated these relationships (Eqs. 4 and 5, respectively) into the model of Wang et al. [32] under two restrictive assumptions. First, we held the search and control costs associated with any given level of invasion constant, that is, we held the parameter values in Eqs. 4 and 5 constant during simulations. Of course, search and control costs vary over time [57] and explicit representation of appropriate trends, with the associated uncertainty, would allow the model to provide a richer context within which to make management decisions. Second, we assumed that land managers knew exactly when the invasion had reached the control threshold on their land and would begin control as soon as this threshold had been reached. Of course, land managers lack perfect knowledge about extent of invasion and make control decisions based on a variety of different incentives [51]. This potentially creates a mosaic of controlled and uncontrolled areas, thus increasing the likelihood of re-invasion from uncontrolled neighboring lands [106]. We easily could represent local differences in invasion awareness and control thresholds in our model, based on appropriate hypotheses regarding differences in intensity of management (invasion awareness) and attitudes toward control (control threshold) [84], [107]. Studies encouraging private landowners to begin monitoring their lands before invasion actually is detected [51] could produce data that would compliment the FIA data collected by U.S. Forest Service in terms of providing a useful source of information for model parameterization.

The quantitative framework we have described in the present paper, unlike that of previous bioeconomic assessments of the spread of invasive species [51], [58], [70], [80], explicitly represents the spatial heterogeneity associated with economic impacts. We agree with Walters that the value of modeling in fields like ecology and natural resource management is not to make precise predictions, but rather to provide clear caricatures of nature against which to test and expand experience [108]. Restrictive assumptions notwithstanding, we believe our model is a useful caricature of the spatial-temporal dynamics of the bioeconomic impacts of invasive terrestrial plants allowing a more integrated approach to evaluating the ecological and economic efficacy of alternative management strategies.
